# 1728. Immune Responses to Cross-Reactive Serotypes 6C and 15C After 20-Valent Pneumococcal Conjugate Vaccine in Infants

**DOI:** 10.1093/ofid/ofad500.1560

**Published:** 2023-11-27

**Authors:** Noor Tamimi, Mary J Kline, Kimberly J Center, Allison Thompson, Gary Baugher, Jelena Drozd, Ingrid L Scully, Peter Giardina, James Trammel, Lanyu Lei, Yahong Peng, Daniel Scott, William C Gruber, Wendy Watson

**Affiliations:** Pfizer Inc, Collegeville, Pennsylvania; Pfizer Inc, Collegeville, Pennsylvania; Pfizer, Collegeville, Pennsylvania; Pfizer Inc, Collegeville, Pennsylvania; Pfizer Inc, Collegeville, Pennsylvania; Pfizer Inc, Collegeville, Pennsylvania; Pfizer Vaccine Research and Development, Pearl River, New York; Pfizer Vaccine Research and Development, Pearl River, New York; Pfizer Inc, Collegeville, Pennsylvania; Pfizer Inc, Collegeville, Pennsylvania; Pfizer Inc, Collegeville, Pennsylvania; Pfizer, Collegeville, Pennsylvania; Pfizer, Collegeville, Pennsylvania; Pfizer Vaccine Research and Development, Pearl River, New York

## Abstract

**Background:**

The 20-valent pneumococcal conjugate vaccine (PCV20) was developed to expand protection against pneumococcal disease. PCV20 contains conjugates for serotype 6A, which is also in 13-valent PCV (PCV13) and appears to have had a clinical impact on 6C disease, and the additional serotype 15B. An exploratory assessment of cross-reactive functional antibodies to serotypes 6C and 15C elicited by PCV20 in two phase 3 infant studies is described.

**Methods:**

Two randomized phase 3 studies evaluated immunogenicity and safety of PCV20 relative to PCV13 in infants receiving a 4-dose series (3 infant doses and a toddler dose; NCT04382326) or a 3-dose series (2 infant doses and a toddler dose; NCT04546425). In each study, immunoglobulin G (IgG) concentrations and opsonophagocytic activity (OPA) titers to serotypes 6C and 15C were measured in a randomly selected subset of infants 1 month after the infant doses, and before and 1 month after the toddler dose. IgG geometric mean concentrations, OPA geometric mean titers (GMTs), and percentage of participants with predefined IgG concentrations, and OPA titers greater than or equal to the lower limit of quantitation were calculated with 95% confidence intervals.

**Results:**

PCV20 elicited IgG and OPA responses to serotype 6C that were generally similar for most endpoints to PCV13 after the 3 infant and toddler doses in the 4-dose series study and after the toddler dose in the 3-dose series study. PCV20 also elicited both IgG and OPA responses to 15C after the infant and toddler doses that were well differentiated from the PCV13 group in both studies. After the toddler dose, OPA GMTs to serotype 6C in the PCV20 and PCV13 groups were 422 and 995, respectively, following a 3-dose series, and 824 and 1090 following a 4-dose series. OPA GMTs to serotype 15C were 67 and 38 and 117 and 38 at the same timepoint in the PCV20 and PCV13 groups, respectively.

**Conclusion:**

PCV20 elicits cross-reactive IgG and cross-functional OPA responses to serotypes 6C and 15C. The responses to serotype 6C after PCV20 are generally similar to those of PCV13. The epidemiology of disease due to the vaccine-type and vaccine-related serotypes will be monitored after introduction of PCV20 into infant immunization programs.

Funded by Pfizer Inc

**Disclosures:**

**Noor Tamimi, MD**, Pfizer: Employee|Pfizer: Stocks/Bonds **Mary J. Kline, MD**, Pfizer Inc: Employee|Pfizer Inc: Stocks/Bonds **Kimberly J. Center, M.D.**, Pfizer Inc: Employee|Pfizer Inc: Stocks/Bonds **Allison Thompson, MD**, Pfizer Inc: Employee|Pfizer Inc: Stocks/Bonds **Gary Baugher, PharmD**, Pfizer Inc: Employee|Pfizer Inc: Stocks/Bonds **Jelena Drozd, MS**, Pfizer Inc: Employee|Pfizer Inc: Stocks/Bonds **Ingrid L. Scully, PhD**, Pfizer Inc: Employee|Pfizer Inc: Stocks/Bonds **Peter Giardina, PhD**, Pfizer Inc: Employee|Pfizer Inc: Stocks/Bonds **James Trammel, MS**, Pfizer Inc: Employee|Pfizer Inc: Stocks/Bonds **Lanyu Lei, PhD**, Pfizer Inc: Employee|Pfizer Inc: Stocks/Bonds **Yahong Peng, PhD**, Pfizer Inc: Employee|Pfizer Inc: Stocks/Bonds **Daniel Scott, MD**, Pfizer Inc: Employee|Pfizer Inc: Stocks/Bonds **William C. Gruber, MD**, Pfizer, Inc.: Employee|Pfizer, Inc.: Stocks/Bonds **Wendy Watson, MD**, Pfizer Inc: Employee|Pfizer Inc: Stocks/Bonds

